# Respiratory Virus Surveillance Among Children with Acute Respiratory Illnesses — New Vaccine Surveillance Network, United States, 2016–2021

**DOI:** 10.15585/mmwr.mm7140a1

**Published:** 2022-10-07

**Authors:** Ariana Perez, Joana Y. Lively, Aaron Curns, Geoffrey A. Weinberg, Natasha B. Halasa, Mary Allen Staat, Peter G. Szilagyi, Laura S. Stewart, Monica M. McNeal, Benjamin Clopper, Yingtao Zhou, Brett L. Whitaker, Elizabeth LeMasters, Elizabeth Harker, Janet A. Englund, Eileen J. Klein, Rangaraj Selvarangan, Christopher J. Harrison, Julie A. Boom, Leila C. Sahni, Marian G. Michaels, John V. Williams, Gayle E. Langley, Susan I. Gerber, Angela Campbell, Aron J. Hall, Brian Rha, Meredith McMorrow, New Vaccine Surveillance Network Collaborators, New Vaccine Surveillance Network Collaborators, Bonnie Strelitz, Kirsten Lacombe, Mary Moffatt, Jennifer Schuster, Chelsea Rohlfs, Miranda Howard, Yesenia Romero, James Chappell, Pedro A. Piedra, Vasanthi Avadhanula, Wende Fregoe, Christina Albertin, Robert H. Hickey, Judith M Martin

**Affiliations:** ^1^Division of Viral Diseases, National Center for Immunization and Respiratory Diseases, CDC; ^2^General Dynamics Information Technology, Inc., Falls Church, Virginia; ^3^Department of Pediatrics, University of Rochester School of Medicine & Dentistry, Rochester, New York; ^4^Department of Pediatrics, Vanderbilt University Medical Center, Nashville, Tennessee; ^5^Department of Pediatrics, University of Cincinnati College of Medicine, Cincinnati, Ohio; ^6^Division of Infectious Diseases, Cincinnati Children’s Hospital, Cincinnati, Ohio; ^7^University of California at Los Angeles, Los Angeles, California;^ 8^IHRC Inc, Atlanta, Georgia; ^9^Department of Pediatrics, Seattle Children’s Hospital, Seattle, Washington; ^10^Department of Pathology and Laboratory Medicine, Children’s Mercy, Kansas City, Missouri; ^11^Texas Children’s Hospital, Houston, Texas; ^12^Baylor College of Medicine, Houston, Texas; ^13^Department of Pediatrics, University of Pittsburgh School of Medicine, Pittsburgh, Pennsylvania; ^14^UPMC Children’s Hospital of Pittsburgh, Pittsburgh, Pennsylvania.; Seattle Children’s Hospital, Seattle, Washington; Seattle Children’s Hospital, Seattle, Washington; Children’s Mercy, Kansas City, Missouri; Children’s Mercy, Kansas City, Missouri; College of Medicine, University of Cincinnati, Cincinnati, Ohio; College of Medicine, University of Cincinnati, Cincinnati, Ohio; Vanderbilt University Medical Center, Nashville, Tennessee; Vanderbilt University Medical Center, Nashville, Tennessee; Baylor College of Medicine, Houston, Texas; Baylor College of Medicine, Houston, Texas; School of Medicine & Dentistry, University of Rochester, Rochester, New York; School of Medicine & Dentistry, University of Rochester, Rochester, New York; UPMC Children’s Hospital of Pittsburgh, Pittsburgh, Pennsylvania; UPMC Children’s Hospital of Pittsburgh, Pittsburgh, Pennsylvania

The New Vaccine Surveillance Network (NVSN) is a prospective, active, population-based surveillance platform that enrolls children with acute respiratory illnesses (ARIs) at seven pediatric medical centers. ARIs are caused by respiratory viruses including influenza virus, respiratory syncytial virus (RSV), human metapneumovirus (HMPV), human parainfluenza viruses (HPIVs), and most recently SARS-CoV-2 (the virus that causes COVID-19), which result in morbidity among infants and young children (*1*–*6*). NVSN estimates the incidence of pathogen-specific pediatric ARIs and collects clinical data (e.g., underlying medical conditions and vaccination status) to assess risk factors for severe disease and calculate influenza and COVID-19 vaccine effectiveness. Current NVSN inpatient (i.e., hospital) surveillance began in 2015, expanded to emergency departments (EDs) in 2016, and to outpatient clinics in 2018. This report describes demographic characteristics of enrolled children who received care in these settings, and yearly circulation of influenza, RSV, HMPV, HPIV1–3, adenovirus, human rhinovirus and enterovirus (RV/EV),* and SARS-CoV-2 during December 2016–August 2021. Among 90,085 eligible infants, children, and adolescents (children) aged <18 years^†^ with ARI, 51,441 (57%) were enrolled, nearly 75% of whom were aged <5 years; 43% were hospitalized. Infants aged <1 year accounted for the largest proportion (38%) of those hospitalized. The most common pathogens detected were RV/EV and RSV. Before the emergence of SARS-CoV-2, detected respiratory viruses followed previously described seasonal trends, with annual peaks of influenza and RSV in late fall and winter (*7*,*8*). After the emergence of SARS-CoV-2 and implementation of associated pandemic nonpharmaceutical interventions and community mitigation measures, many respiratory viruses circulated at lower-than-expected levels during April 2020–May 2021. Beginning in summer 2021, NVSN detected higher than anticipated enrollment of hospitalized children as well as atypical interseasonal circulation of RSV. Further analyses of NVSN data and continued surveillance are vital in highlighting risk factors for severe disease and health disparities, measuring the effectiveness of vaccines and monoclonal antibody–based prophylactics, and guiding policies to protect young children from pathogens such as SARS-CoV-2, influenza, and RSV.

During December 1, 2016–August 31, 2021, NVSN enrolled children aged <18 years in inpatient and ED settings at seven surveillance sites (Supplementary Table 1, https://stacks.cdc.gov/view/cdc/121550). Children were eligible for enrollment if they had an illness duration of <14 days, were enrolled within 48 hours of admission (inpatient only), had at least one qualifying ARI sign or symptom (e.g., apnea, cough, earache, fever, myalgia, nasal congestion, runny nose, sore throat, vomiting after coughing, shortness of breath [rapid or shallow breathing], wheezing, or apparent life-threatening event or brief resolved unexplained event), and resided in a surveillance site area.^§^ Children were excluded if they had a known nonrespiratory cause for hospitalization, had fever and neutropenia from chemotherapy, were admitted <5 days after a previous hospitalization, were transferred from another hospital after an admission of >48 hours, were a newborn who had never been discharged home from the hospital, or had previously enrolled in this study <14 days before their current visit or hospitalization. Children could be enrolled in inpatient units ≥5 days per week and in the ED ≥4 days per week for ≥6 hours per day.

Outpatient clinic enrollment began in November 2018, with enrollment limited to children aged <2 years and testing for RSV only. Enrollment and testing were later expanded to include children aged <18 years and multipathogen testing.^¶^ Outpatient enrollment was paused during May–October 2019, and weekly enrollment targets of approximately 150 patients were required before July 2020. Outpatient eligibility and exclusion criteria differed slightly from that of other clinical settings.** Beginning in April 2020, outpatient surveillance was expanded in Houston, Texas to include drive-through testing for SARS-CoV-2 (*9*). Data in this report are summarized by highest level of care received by each child, irrespective of the child’s enrollment setting.

Midturbinate (MT) nasal or oropharyngeal (OP) specimens were obtained using flocked swabs; if both nasal and OP swabs were collected, they were combined and placed in universal transport medium. A tracheal aspirate was accepted as an alternative specimen for patients who were intubated. Among patients from whom research MT nasal and OP or tracheal aspirate specimens could not be obtained, clinically obtained respiratory specimens were salvaged.^††^ Specimens were transported to the laboratory at each site and stored at a temperature of 35.6°F–46.4°F (2°C–8°C) until they were processed (within 72 hours). Specimen aliquots were subsequently frozen at −94°F (−70°C) or lower. Specimens underwent molecular testing at each study site for respiratory pathogens including RSV, influenza, HMPV, HPIV1–3, RV/EV, and adenovirus. SARS-CoV-2 surveillance and associated testing methodologies^§§^ began in 2020.^¶¶^ Molecular diagnostic assay methods used for respiratory pathogens varied by site (Supplementary Table 2, https://stacks.cdc.gov/view/cdc/121551) (Supplementary Table 3, https://stacks.cdc.gov/view/cdc/121552). All assays met CDC-sponsored proficiency testing standards.

Pearson’s chi-square tests compared the percentage of positive results during the 2020–2021 season against previous seasons combined, among inpatients and those treated in the ED. All analyses were performed using SAS software (version 9.4; SAS Institute). Informed consent was obtained from a parent or legal guardian of eligible children before conducting a standardized parent or guardian interview; medical chart review; and collection, testing, and storage of respiratory specimens. Assent from eligible children was obtained at each site, according to local regulations. This study was reviewed and approved by the institutional review boards at each of the seven study sites.***

During December 2016–August 2021, a total of 90,085 eligible children with ARI were identified and 51,441 (57%) were enrolled. Within the highest clinical care setting received, enrolled children included 22,093 (43%) inpatients, 23,145 (45%) patients evaluated in the ED, and 6,203 (12%) evaluated in outpatient clinics (Table 1). Among all enrolled children, 38,267 (74%) were aged <5 years, 15,986 (42%) of whom were aged <1 year. The majority of enrolled children (55%) were male; 32% were non-Hispanic Black or African American (Black), 31% were non-Hispanic White (White) and 27% were Hispanic or Latino (Hispanic) children. Among hospitalized children, 8,280 (38%) were aged <1 year, 12,623 (57%) were male, and 9,042 (41%) were White.

**TABLE 1:** Demographic characteristics of enrolled children and adolescents aged <18 years, by highest level of care setting — New Vaccine Surveillance Network, United States, December 2016–August 2021*^,^^†^

Characteristic	Highest care level setting, no. (column %)			
	All	Inpatient	ED^†^	Outpatient^§^
**Overall**	**51,441 (100.0)**	**22,093 (100.0)**	**23,145 (100.0)**	**6,203 (100.0)**
**Age group**				
0–11 mos	**15,986 (31.1)**	8,280 (37.5)	6,150 (26.6)	1,556 (25.1)
12–23 mos	**10,339 (20.1)**	4,023 (18.2)	4,997 (21.6)	1,319 (21.3)
24–59 mos	**11,942 (23.2)**	4,356 (19.7)	6,433 (27.8)	1,153 (18.6)
5–17 yrs	**13,174 (25.6)**	5,434 (24.6)	5,565 (24.0)	2,175 (35.1)
**Sex**				
Male	**28,473 (55.4)**	12,623 (57.1)	12,639 (54.6)	3,211 (51.8)
Female	**22,967 (44.7)**	9,470 (42.9)	10,506 (45.4)	2,991 (48.2)
Unknown	**1 (0.0)**	0 (—)	0 (—)	1 (0.0)
**Race or ethnicity**				
Black or African American, non-Hispanic	**16,582 (32.3)**	5,249 (23.8)	9,879 (42.7)	1,454 (23.4)
Hispanic or Latino	**13,771 (26.8)**	5,476 (24.8)	6,012 (26.0)	2,283 (36.8)
Other	**4,615 (9.0)**	2,135 (9.7)	1,863 (8.1)	617 (10.0)
White, non-Hispanic	**16,028 (31.2)**	9,042 (40.9)	5,214 (22.5)	1,772 (28.6)
Unknown	**445 (0.8)**	191 (0.7)	177 (0.8)	77 (1.2)

**Abbreviations:** ED = emergency department; RSV = respiratory syncytial virus.

* Among ED surveillance sites, enrollment was restricted to children aged <5 years during the following periods: Seattle during December 2016–June 2017, November 2017–June 2018, November 2018–June 2019, and December 2019–March 2020; Pittsburgh during December 2016–June 2018, November 2018–June 2019, and December 2019–March 2020; Kansas City during December 2016–June 2017, November 2017–June 2018, and November 2018–June 2019.

^†^ Outpatient enrollment began in November 2018, paused during May–October 2019, and resumed with enrolled children aged <2 years during November 2018–July 2020; RSV testing was prioritized during November 2018–April 2019.

Across all settings, 32,259 (63%) specimens had at least one viral pathogen detected, 4,492 (9%) had more than one viral pathogen detected, and 19,182 (37%) had no viral pathogen detected. The pathogens most frequently detected were RV/EV (14,906; 31%) and RSV (8,461; 17%) (Table 2). Total proportions for each virus varied by setting; RSV was detected most frequently in inpatient settings (24%), influenza in EDs (11%), and RV/EV in outpatient clinics (39%). During the COVID-19 pandemic period (March 2020–August 31, 2021), 1,171 (7%) children received a positive SARS-CoV-2 test result, 411 (35%) of whom were outpatients. During the 2020–2021 season (September 15, 2020–August 31, 2021), lower total proportions of test results were positive for seasonal viruses compared with previous seasons combined among inpatient and ED settings, except for HPIV1–3 (8%) and RV/EV (36%) (p<0.001). Enrollment during December 2016–February 2020, peaked in inpatient and ED settings, with concurrent peaks in RSV and influenza detections. Other viruses such as adenovirus and HMPV circulated throughout this period, but smaller peaks occurred later in winter and early spring (Figure). After onset of the COVID-19 pandemic in March 2020, inpatient and ED enrollment did not follow previously observed seasonal patterns; enrollment and virus circulation during winter months of 2020 was lower than expected and a distinct peak in RSV circulation and overall enrollment occurred during summer months of 2021.

**TABLE 2:** Respiratory virus detections* among enrolled children and adolescents aged <18 years, by highest level of care setting and surveillance season^†^ — New Vaccine Surveillance Network, United States, December 2016–August 2021

Characteristic	Viral pathogen, no. (column %)						
	Adenovirus	Influenza	HMPV	HPIV1–3	RSV	RV/EV	SARS-CoV-2^§^
	N = 48,859	N = 49,045	N = 48,859	N = 48,859	N = 49,994	N = 48,847	N = 16,386
**Highest care setting**							
Inpatient	**872 (4.1)**	**1,122 (5.2)**	**930 (4.3)**	**1,081 (5.0)**	**5,085 (23.7)**	**6,551 (30.6)**	**377 (7.1)**
ED	**1,622 (7.2)**	**2,451 (10.8)**	**960 (4.2)**	**1,903 (8.4)**	**2,936 (12.9)**	**6,493 (28.6)**	**383 (5.9)**
Outpatient^¶^	**122 (2.6)**	**75 (1.5)**	**47 (1.0)**	**195 (4.1)**	**440 (7.6)**	**1,862 (39.3)**	**411 (9.0)**
**Surveillance season**							
2016–2017	600 (6.0)	797 (8.0)	565 (5.7)	696 (7.0)	1,803 (18.1)	2,888 (29.1)	NA
2017–2018	538 (6.3)	856 (10.1)	451 (5.3)	599 (7.0)	1,512 (17.8)	2,618 (30.8)	NA
2018–2019	643 (6.8)	816 (8.6)	524 (5.5)	784 (8.2)	1,859 (17.9)	3,023 (31.8)	NA
2019–2020	458 (5.1)	1,169 (12.7)	368 (4.1)	166 (1.8)	1,845 (20.0)	2,108 (23.4)	258 (6.8)
2020–2021	377 (3.2)	10 (0.1)	29 (0.3)	934 (7.9)	1,442 (12.1)	4,269 (35.9)	913 (7.3)
**All years**	**2,616 (5.4)**	**3,648 (7.4)**	**1,937 (4.0)**	**3,179 (6.5)**	**8,461 (16.9)**	**14,906 (30.5)**	**1,171 (7.1)**

**Abbreviations**: ED = emergency department; HMPV = human metapneumovirus; HPIV1–3 = human parainfluenza virus types 1–3; NA = not applicable; RSV = respiratory syncytial virus; RV/EV = rhinovirus and enterovirus.

* Respiratory virus detection results are from research swab specimens that underwent molecular testing, except for SARS-CoV-2, which included both research and clinical specimens to most accurately represent viral detections across surveillance years. Denominators for positivity rates are pathogen-specific.

^†^ Surveillance seasons during 2016–2017 were December 1, 2016–November 30, 2017; 2017–2018: December 1, 2017–October 31, 2018; 2018–2019: November 1, 2018–October 31, 2019; 2019–2020: November 1, 2019–September 14, 2020; 2020–2021: September 15, 2020–August 31, 2021.

^§^ SARS-CoV-2 was first detected in 2020, test results for SARS-CoV-2 reported in this table are from the pandemic period (March 2020–August 2021); surveillance years 2016–2017 through 2018–2019 were not applicable.

^¶^ Outpatient data were not included for seasons 2016–2017 and 2017–2018 because outpatient enrollment did not begin until November 2018.

**FIGURE:**
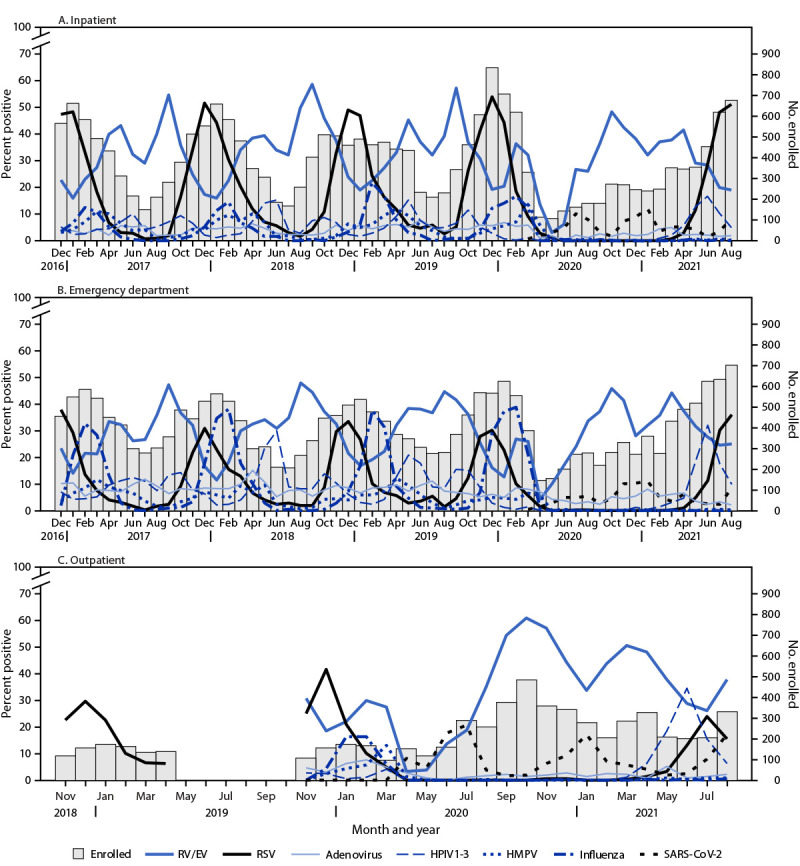
Respiratory virus detections among enrolled children and adolescents aged <18 years with research tested specimens, by highest level of care in inpatient (A), emergency department (B), and outpatient (C) settings — New Vaccine Surveillance Network, United States, December 2016–August 2021*^,^^†^^,^^§^ **Abbreviations**: ED = emergency department; HPMV = human metapneumovirus; HPIV1–3 = human parainfluenza virus types 1–3; RSV = respiratory syncytial virus; RV/EV = rhinoviruses and enteroviruses. * Outpatient enrollment began in November 2018, paused during May–October 2019, and resumed with enrolled children aged <2 years during November 2018–July 2020; RSV testing was prioritized during November 2018–April 2019. ^†^ SARS-CoV-2 detections only included research positive test results for consistency across pathogens; therefore, total detections are underrepresented. ^§^ Surveillance was paused at these sites during the COVID-19 pandemic: Cincinnati (inpatient: March 25–30, 2020; ED: March 24–30, 2020; and outpatient: March 25, 2020); Seattle (outpatient: March 2–12, 2020 and March 13–31, 2020); Houston (inpatient, ED, and outpatient: March 23–31, 2020); Kansas City (inpatient: March 18–29, 2020; ED: March 18–28, 2020; outpatient: March 18–31, 2020); and Pittsburgh (inpatient and ED: March 22–29, 2020 and outpatient: March 13–31, 2020).

## Discussion

During 2016–2021, approximately 51,000 children with ARI were prospectively enrolled in NVSN. Nearly 75% of enrolled children were aged <5 years, and children aged <1 year accounted for approximately one third of those hospitalized, consistent with previous studies among this age group (*1*–*5*). NVSN enrollees were racially and ethnically diverse, with nearly one third being Black children followed by slightly lower percentages of White and Hispanic children. Before the COVID-19 pandemic, seasonal patterns of respiratory virus circulation followed previously described trends, including annual peaks of influenza and RSV during late fall and winter months (*7*,*8*). RV/EV and RSV were the most frequently detected viruses in children in all settings; however, by setting, RSV was more commonly detected among hospitalized children than it was in ED or outpatient clinics. During the 2020–2021 season, the total proportion of seasonal respiratory viruses was lower than that during previous seasons for all except HPIV1–3 and RV/EV. These declines support previous studies, which postulated that community mitigation measures (e.g., school and child care facility closures) during the COVID-19 pandemic had contributed to decreased circulation of respiratory viruses such as influenza and RSV (*10*). Pandemic period enrollment did not follow seasonal trends, with a notable increase in inpatient and ED enrollments during summer months of 2021. This increase was largely associated with the return of RSV after nearly a year without community circulation.

The findings in this report are subject to at least four limitations. First, NVSN data are limited to enrolled and consented participants who might not be representative of all children seeking care at a healthcare facility. Second, although NVSN surveillance sites are located across the United States, they might not be representative of the entire country. Third, outpatient clinic surveillance differed from the more consistent inpatient and ED surveillance in several ways, including a later start date, prioritized RSV testing during first year of enrollment, paused enrollment during May 2019–October 2019, and age restrictions in several sites, making it difficult to establish trends during the surveillance period. Finally, new approaches to outpatient surveillance (e.g., drive-through clinics) were implemented during the COVID-19 pandemic, which affected enrollment and proportion of positive SARS-CoV-2 test results in this setting.

Prospective ARI surveillance in NVSN measured seasonal trends in respiratory virus circulation before and during the COVID-19 pandemic. These data have the potential to estimate population-based rates of SARS-CoV-2, RSV, and other respiratory virus hospitalizations, ED, and outpatient visits. Further analyses of NVSN data and continued surveillance are vital in highlighting risk factors for severe disease and health disparities, measuring the effectiveness of vaccines and monoclonal antibody–based prophylactics, and guiding policies to protect young children from pathogens such as SARS-CoV-2, influenza, and RSV.

**Summary**

**What is already known about this topic?**

Acute respiratory illness (ARI) caused by viruses including respiratory syncytial virus (RSV) and SARS-CoV-2 (the virus that causes COVID-19) results in pediatric morbidity.

**What is added by this report?**

Rhinovirus and enterovirus and RSV were the most frequently detected viruses among children enrolled in the New Vaccine Surveillance Network during 2016–2021 through inpatient, outpatient, and emergency department settings. Throughout the COVID-19 pandemic, respiratory viruses exhibited uncharacteristic seasonality, with lower-than-expected circulation during April 2020–May 2021, and atypical RSV circulation and inpatient enrollment in summer 2021.

**What are the implications for public health practice?**

Continued ARI surveillance is critical as vaccines and therapeutics are introduced to protect children from SARS-CoV-2 and RSV to elucidate risk factors, health disparities, and to guide prevention policies.

## Footnotes

* Diagnostic assays used for RV/EV might detect only rhinovirus, only enterovirus, or rhinovirus and enterovirus combined.

† Surveillance sites in Kansas City, Pittsburgh, and Seattle restricted ED enrollment primarily to children aged <5 years annually during winter–spring (2016–2019).

§ The seven U.S. pediatric medical center NVSN surveillance sites were in Cincinnati, Ohio; Houston, Texas; Kansas City, Missouri; Nashville, Tennessee; Pittsburgh, Pennsylvania; Rochester, New York; and Seattle, Washington.

¶ Outpatient enrollment of children aged <2 years began in November 2018 and expanded to enroll children aged <18 years during July–August 2020; testing in outpatient settings expanded from RSV alone to multipanel testing in November 2019.

** In general, children were eligible for enrollment if they met some of the same criteria as inpatient and ED patients, which included apnea, myalgia, vomiting after coughing, or apparent life-threatening event or brief resolved unexplained event. Outpatient exclusion criteria also differed; children were excluded if they were seen at an outpatient, inpatient, or ED setting <5 days after an acute respiratory illness, or had been enrolled as outpatients within the previous 4 days.

†† Surveillance sites were provided with instructions on how to properly obtain respiratory specimens and a list of specimen types that might be acceptable to use when a clinical salvage was the only option (i.e., tracheal aspirate, MT, OP, bronchoalveolar lavage, sputum, or nasal wash). Investigators were asked to consult with CDC to determine acceptability of clinically salvaged specimens.

§§ SARS-CoV-2 research testing began during March–April 2020 and was not implemented systematically during the onset of the pandemic because of suspension of enrollment and surveillance activities for 1–3 weeks across sites.

¶¶ Each site performed retrospective SARS-CoV-2 testing on respiratory specimens from children who were enrolled and had specimens collected beginning either January 1 or February 1, 2020.

*** 45 C.F.R. part 46; 21 C.F.R. part 56.
